# Short and Long-Term Cardiovascular Sequelae after SARS-CoV-2 Infection: A Narrative Review Focusing on Athletes

**DOI:** 10.3390/v15020493

**Published:** 2023-02-10

**Authors:** Sara Monosilio, Silvia Prosperi, Maria Rosaria Squeo, Stefano Spataro, Antonio Spataro, Viviana Maestrini

**Affiliations:** 1 Institute of Sport Medicine and Science of Rome, Italian National Olympic Committee, CONI, 00197 Rome, Italy; 2 Department of Clinical Internal, Anesthesiological and Cardiovascular Sciences, Sapienza University of Rome, 00161 Rome, Italy; 3 Department of Clinical Internal Medicine, Tor Vergata University of Rome, 00133 Rome, Italy

**Keywords:** COVID-19, SARS-CoV-2 infection, cardiovascular sequelae, athletes, myocardial injury, myocarditis, pericarditis, cardiovascular magnetic resonance (CMR), cardiopulmonary exercise test (CPET)

## Abstract

Cardiovascular (CV) involvement after severe acute respiratory syndrome coronavirus 2 (SARS-CoV2) infection was found to be frequent among the general population, especially in the pre-vaccination era, and particularly for hospitalized patients or those who experienced a more severe course of the disease. The spectrum of CV disease varies; however, acute myocarditis is particularly fearsome for the athletic population due to the possible associated risk of malignant arrhythmias during training. Alarming percentages of CV injuries, even in young and healthy athletes with a benign course of the disease, arose from a few initial studies limited to case series. Subsequent single-center studies and larger observational registries reported a lower prevalence of SARS-CoV2 CV involvement in athletes. Studies showing the occurrence of CV adverse events during follow-up periods are now available. The objective of our narrative review is to provide an updated summary of the literature on CV involvement after coronavirus disease 2019, both in the early post-infection period and over a longer period of time, with a focus on athletic populations.

## 1. Introduction

It has been nearly three years since the World Health Organization declared the coronavirus disease 2019 (COVID-19) pandemic outbreak in March 2020, caused by the diffusion of Severe Acute Respiratory Syndrome Coronavirus 2 (SARS-CoV2). The pandemic caused millions of deaths, and concerns about COVID-19 sequelae have been raised. Cardiovascular (CV) involvement is frequent in patients suffering from COVID-19, with various manifestations ranging from asymptomatic myo-pericarditis to arrhythmias, pulmonary emboli, myocardial infarction, and cardiogenic shock [1,2,3,4]. The COVID-19 pandemic has had a variety of consequences, even in the world of sports. Firstly, at the beginning of the pandemic, sports competitions at the national and international level, including the 2020 Olympic games, were withdrawn or postponed. Secondly, the possible CV involvement of COVID-19 could have a cascade effect on sport participation. The CV involvement during an infection and possibly related sequelae generated concerns not only in the general population but also in the athletic one, particularly during the pre-vaccination era. Over the past three years, a wide number of studies have been published on the short-term CV sequelae in athletes, while less is known regarding possible long-term effects on the CV system. This narrative review focuses on CV sequelae in athletes. The overall aim of the review is to provide a summary of studies on short-term sequelae and acute or post-infection CV involvement in athletic populations; a specific objective was to focus on those studies with a longer follow-up, which are currently becoming available in the literature.

## 2. Methods

A detailed literature search of articles and reports on cardiovascular involvement and sequelae after SARS-CoV-2 infection in athletes was conducted on PUBMED/MEDLINE and Cochrane electronic medical databases. The following medical search (MeSH) terms were used: COVID-19, SARS-CoV-2, athletes, myocardial injury, myocarditis, pericarditis, cardiovascular complication, cardiovascular sequelae, cardiac magnetic resonance (CMR), and myocardial damage. We included published prospective and retrospective studies conducted both in single and multiple centers and on populations of both professional and non-professional athletes. Only English language works were included in this narrative review. Meta-analysis and systematic reviews were excluded. The research covered a period between April 2020 and November 2022.

## 3. Discussion

### 3.1. COVID-19 and the Cardiovascular System

During the first wave of the pandemic, it became clear that pre-existing CV diseases and CV involvement worsened the course of the infection, especially in hospitalized patients [5,6,7,8,9]. CV involvement can occur during the infection and after the recovery, especially for subjects who experienced a more severe course of the disease [8,9]. Many studies demonstrated that risk factors such as hypertension, diabetes, and a history of cardio/cerebrovascular diseases were associated with higher mortality in COVID-19 cohorts [10,11,12,13].

SARS-CoV2 affects the CV system through several mechanisms: (1) direct virus effect on cardiomyocytes and endothelial cells due to the great expression of angiotensin-converting enzyme 2 receptors, used by the virus to enter human cells, in cardiovascular system tissues; (2) hypercoagulability because of the interaction between the virus and endothelial cells and also due to the inflammatory status; (3) cytokine release, which could evolve into a “cytokine storm” during exaggerated immune responses; and (4) hypoxia as a consequence of pneumonia or acute respiratory distress syndrome [14]. As a result, CV manifestations could be heterogeneous, including myocardial infarction, arrhythmias, heart failure, thromboembolism, myocarditis, and myocardial injury [2,15].

### 3.2. COVID-19 and Cardiovascular Involvement in Athletes

The first evidence of CV involvement in athletes was described during the first wave of the pandemic and initially came from case series. One of the first reports showed an alarming prevalence of 15% of myocarditis, detected by cardiac magnetic resonance (CMR), in a cohort of 26 athletes who were asymptomatic and without other abnormalities [16]. Brito et al. also described 39% pericardial involvement, with 12.5% and 16.7% concomitant and isolated myocardial involvement, respectively, at CMR and transthoracic echocardiography (TTE) in a cohort of 54 collegiate athletes [17]. These initial two case series reported the highest percentages of athletes’ COVID-19 myocardial and pericardial involvement, which were not confirmed by the following studies. On the contrary, other reports on small cohorts did not find evidence of definite cardiovascular involvement after COVID-19 [18,19,20,21,22,23,24].

Later, some studies reported the possible occurrence of myocarditis and pericarditis after SARS-CoV-2 infection with a lower prevalence [25,26,27,28,29,30,31]. Starekova et al. performed a retrospective study on 145 student-athletes who underwent blood tests, TTE, and CMR after COVID-19, reporting 1.4% of myocarditis [25]. A prospective study on 147 highly trained athletes recovering from SARS-CoV2 infection and undergoing Troponin I, an electrocardiogram (ECG), and a CMR before return to play (RTP) found 1.4% of athletes met the criteria for a diagnosis of myocarditis [26].

All the studies reported so far have applied a similar protocol based on ECG, roponin, and TTE. Results from studies applying different RTP protocols included exercise testing or cardiopulmonary exercise testing (CPET). Maestrini et al. screened a cohort of 47 Olympic athletes with previous SARS-CoV-2 disease based on the RTP protocol recommended during the first phase of the pandemic (including a blood test, ECG, TTE, CPET, and 24-h Holter ECG monitoring). CMR was also included in this study. Acute myocarditis was detected in 2% of the athletes, and those athletes presented with symptoms, new exercise-induced premature ventricular contractions (PVCs), and increased troponin T [28]. In a subsequent study on 219 athletes, at the RTP evaluation, 9.5% of athletes presented with uncommon PVCs at CPET or Holter monitoring, but the prevalence of myocarditis was 0.9% [29]. Interestingly, all athletes had good performances after recovering from the infection without functional limitations.

Cavigli et al. found 3.3% of cardiac abnormalities in a cohort of 90 professional and non-professional athletes from a single-center study recovering from COVID-19 (one myo-pericarditis and two pericarditis) [30].

Regardless of the protocol applied, all the mentioned studies suffer from several limitations. First, they are single-center, often retrospective, with different interval times between infection and CV evaluation and different methodologies and protocols applied. Later, multicenter registries collected data on a larger scale to confirm the low prevalence of cardiovascular consequences (Table 1). Moulson et al. performed an analysis on 3018 collegiate athletes from the outcome registry for cardiac conditions in athletes (ORCCA) based on ECG, troponin, and TTE (the so-called “triad test”). The diagnosis of myocarditis was divided into definite, probable, and possible categories based on imaging and biomarker criteria, and the overall prevalence was 0.7% of the entire population. In addition, the authors explored the diagnostic role of CMR screening athletes using two different protocols: in one group, CMR was requested based on clinical indication, while in the other, it was performed as a screening tool on all athletes. The authors found that the diagnostic power of CMR for SARS-CoV-2 cardiac involvement was 4.2 times higher for a clinically indicated CMR [12.6% (15/119) vs. 3.0% (6/198)]. Importantly, at multivariate analysis, cardiopulmonary symptoms and abnormal triad tests were predictive of cardiac involvement [32]. Daniels et al. analyzed data on 1597 athletes from 13 universities participating in the Big Ten COVID-19 Cardiac Registry who were recovering from SARS-CoV2 infection and undergoing CV evaluation, which includes a CMR. They showed a prevalence of myocarditis of 2.3% [33]. In contrast to Moulson et al., this study also showed that performing CMR imaging as a screening strategy provided an increased prevalence of cardiac involvement of 7.4-fold and 2.8-fold over a symptoms-driven strategy and the triad test strategy, respectively. However, the clinical significance of isolated positive CMR findings is questionable. Subsequent multicenter studies confirmed a low percentage of cardiac abnormalities at CV evaluation before RTP, finding a prevalence of myocarditis that ranges from 0% to 1.4% [34,35,36,37].

Finally, in a multi-center study focused on 571 junior athletes, the RTP evaluation, including a 12-lead resting ECG, exercise testing, and echocardiography, showed cardiac complications were uncommon and not associated with malignant ventricular arrhythmias [41].

### 3.3. COVID-19 and Cardiovascular Involvement in Athletes: RTP Protocols

With the increasing evidence of possible SARS-CoV-2-related sequelae, different scientific societies have proposed screening protocols for the return to play (RTP) of athletes recovering from the infection, based primarily on expert opinion [42,43,44,45]. The approaches proposed were different and based on different diagnostic algorithms, and they evolved in parallel with advances in pathophysiology understanding and accumulating evidence. Thus, these consensus documents were progressively updated. The American College of Cardiology (ACC) has recently published an updated consensus on the decision pathway for CV sequelae in adults after recovery from COVID-19, including a section dedicated to athletic groups [46]. Previous documents recommended a common approach based on ECG, troponin, and TTE as a screening protocol, the so-called “triad testing” [44,45]. Currently, this approach is reserved only for hospitalized athletes or those who experienced cardio-pulmonary symptoms during the infection, developed new-onset cardio-pulmonary symptoms during the return to physical activity, or had post-acute SARS-CoV2 sequelae [46]. Recently, a European consensus on the RTP in children and junior athletes has been published, recommending a strategy based on the disease severity and the presence of cardiac symptoms to screen athletes who need further CV evaluation after COVID-19 [47]. Athletes who experienced a more severe course of the infection or manifested cardiac symptoms should undergo resting ECG, blood testing, TTE, exercise testing, 24 h Holter ECG monitoring, and CMR if clinically indicated. Individuals with asymptomatic or mild symptoms should be evaluated with an accurate anamnesis and physical examination, and they should be informed about the possibility of CV symptoms occurring during the return to physical activity [47].

### 3.4. Short- and Long-Term Follow-up

The vast majority of the studies were focused on the athletes’ evaluation at the RTP assessment following SARS-CoV-2 infection, with no data on longer follow-up in those athletes with CV involvement. In a few single-center studies, data on longer follow-up in those athletes with myocardial involvement is available, but it is limited to sporadic cases. In such cases, no adverse events after resuming exercise or during competitions were reported [18,24]. The recovery was reported as complete or partially complete, with LGE or arrhythmias persisting (Table 1) [25,28,29,30].

All the registries and multi-center studies on larger cohorts reported results based on clinical follow-up (Table 1). Moulson et al. demonstrated the absence of adverse cardiac events in athletes with definite or probable SARS-CoV2 involvement that were observed for a median of 130 days. Regarding the cohort of positive athletes (median follow-up: 113 days), there was only one (0.3%) resuscitated cardiac arrest, which was probably unrelated to a previous SARS-CoV2 infection since the CMR acquired early after COVID-19 symptoms was negative [32]. Similarly, Chevalier et al. reported over a follow-up period of 289 ± 56 days, with only one case of ventricular tachycardia in an athlete recovered from COVID-19. However, the individual underwent a comprehensive CV evaluation post-infection without any abnormal CV findings [35]. Daniels et al. performed a CMR follow-up in athletes with myocarditis after 9.4 ± 3.1 weeks, finding a complete resolution of abnormalities in 40.7% of the subgroup, while the other 59.3% showed the resolution of oedema with the persistence of LGE [33]. Other registries did not report cardiac adverse events among athletes who underwent cardiac screening and returned to sports competitions [34,37]. Petek et al. conducted a prospective observational study on 3644 athletes from the ORCCA registry focused on persistent or exertional symptoms upon return to exercise after SARS-CoV-2 infection, finding a low prevalence of persistent symptoms (0.12% and 0.06% of athletes complaining of symptoms lasting more than 3 weeks and 12 weeks, respectively). Exertional cardio-pulmonary symptoms were present in 4% of the athletes, the most frequent being shortness of breath and chest pain, and SARS-CoV2 sequelae were diagnosed in 8.8% of athletes with exertional symptoms. Definite or probable SARS-CoV2-related cardiac involvement was diagnosed in 20.8% (5/24) of athletes with exercise-induced chest pain who underwent CMR (three definite pericardial, one definite myo-pericardial, and one probable myo-pericardial), while none of the athletes with other exertional symptoms were diagnosed with cardiac involvement. The authors concluded that SARS-CoV2 cardiac involvement is rare among young competitive athletes and that CV evaluation before RTP has to follow a symptom-based strategy, especially considering chest pain during exercise as a red flag for COVID-19 cardiac consequences [39]. Similarly, Moulson et al. conducted a study on a small cohort of 21 athletes who had persistent or new onset cardio-pulmonary symptoms after COVID-19 and were undergoing a comprehensive CV evaluation with CMR based on clinical indication. No evidence of active inflammatory heart disease was found. Moreover, the athletes performed CPET, showing similar values of peak oxygen consumption (vO_2_) but a lower breathing reserve and, more frequently, abnormal spirometry when compared with a group of COVID-19-negative athletic controls. Thirteen COVID-19-positive athletes underwent CPET at follow-up (4.8 ± 1.9 months), with a resolution or reduction in cardiopulmonary symptoms in 69% of them and a concomitant lower peak heart rate and higher values of peak oxygen consumption [38].

Finally, a more recent study by Petek et al. on 3675 athletes with previous COVID-19 focusing on cardiovascular outcomes at a median follow-up of 1.12 years found 0.6% of definite or probable cardiac involvement (myocardial or myo-pericardial) after the infection and only two adverse cardiac events (0.05%) in the group of COVID-19-positive athletes without SARS-CoV2 CV involvement. One athlete experienced a resuscitated cardiac arrest that was not linked to the previous infection that occurred more than three months earlier. The other athlete had a new onset of atrial fibrillation, possibly related to SARS-CoV2 disease because it occurred less than two weeks before the episode [40].

### 3.5. Limitations and Quality of the Involved Studies

Apart from a few earlier studies, the current literature demonstrates the low prevalence of CV involvement in athletes after COVID-19. However, the studies involved in this narrative review show some limitations. First, most of them are single-center studies with small populations of athletes [16,17,18,19,20,21,23,27,28,38]. Moreover, some works have been conducted retrospectively [20,22,23,24,25,27,31]. There are no randomized controlled trials available. Finally, most works include professional and non-professional athletes with heterogeneous training regimens and cardiovascular involvement during physical activity.

We included eight multi-center studies with larger sample sizes in our narrative review. The vast majority had an observational and prospective design and included a significant number of athletes [32,33,35,36,39,40]. Furthermore, most multi-center registries reported a mid- to long-term clinical follow-up (the longest being 376 ± 125 days) [37], showing the absence or low prevalence of cardiac adverse events in athletes recovering from COVID-19. However, there is a need for prolonged observation and follow-up of COVD-19-positive athletic populations.

### 3.6. Practical Implications and Need for Future Research

Evidence from the available literature, both single- and multi-center observational studies, suggests low percentages of CV involvement and sequelae after SARS-CoV-2 infection among athletic populations, also at more extended follow-up periods. RTP protocols guide the management of professional and non-professional athletes recovering from the infection, but the strategy is still different among countries. Updated protocols have become available based on accumulating evidence and are now mainly based on a symptoms-driven strategy. When evaluating an athlete or individual practicing sports after a SARS-CoV-2 infection, particular attention should be paid to the course of the infection and its severity. After, the presence of CV symptoms, such as chest pain and palpitations, both during the infection and when returning to exercise, need to be considered a red flag for CV involvement and should suggest the need for further investigation with an ECG, laboratory testing, imaging (TTE and even CMR, if necessary), and exercise testing. However, it must be considered that it is still unclear which approach is best for post-infection athletes’ evaluation. Different countries apply different protocols, and trials that compare evaluation approaches have not been conducted but would be beneficial.

## 4. Conclusions

Although initial studies reported alarming percentages of SARS-CoV-2 CV involvement, mainly myocarditis, in young athletes’ cohorts with a benign course, subsequent evidence from large registries disconfirmed it. On the contrary, most studies showed low CV involvement after COVID-19 among young and healthy athletes. At the same time, studies with more extended follow-up periods in athletic populations became available, reporting fewer adverse cardiac events that were not always linked to the previous infection. Even if encouraging observations emerge from the literature, longer follow-up and systematic evaluation are needed to definitively confirm these findings.

## Figures and Tables

**Table 1:** Studies on cardiovascular involvement in athletes after SARS-CoV2 infection.

First Author, Journal, Year	Type of Study	Population	Methods	Timing of Evaluation	Follow-Up	Main Findings
Single-Center Studies						
Rajpal et al. JAMA Cardiology, 2021 [16]	Prospective observational study	26 unselected athletes (mean age 27 ± 1.5 years, 57.7% male).	− ECG, TTE, serum troponin, and CMR performed after COVID-19 recovery	COVID-19 diagnosis-CMR: (11–53 days).	-	− Myocarditis: 15% of athletes (4 cases) − Pericardial involvement: 2 athletes (pericardial effusion along with myocarditis);
Brito et al. Journal of American college of cardiology, 2021 [17]	Cross-sectional study	54 collegiate athletes (mean age 19 years; 85% male).	− Troponin I, ECG, and TTE. − CMR if abnormalities at ECG/TTE. − Control group of COVID-19-negative athletes	COVID-19 diagnosis-CMR: 27 days (range of 22 to 33 days).	-	− Myocarditis: 0% − High troponin I: one subject (6%) − Imaging abnormalities: 56% − Pericardial enhancement + pericardial effusion: 39% − Myo-pericardial abnormalities (GLS reduction, native T1 increase, or both): 12.5% − Isolated myocardial involvement: 16.7% − Myocardial LGE: 2%
Peidro et al. Medicina, 2021 [18]	Prospective observational study	24 international level professional soccer players (median age 27 years, 20–36).	− ECG, TTE, and CMR	-	4 months with participation in national and international competitions	− Cardiac abnormalities: 0% − At 4 months follow-up: 0% cardiac events
Vago et al. Journal of American college of cardiology, 2021 [19]	Observational study	12 professional elite athletes (median age 23 years, 20–23; 10 females).	− Blood testing (troponin T, pro-BNP, and CRP) and CMR after recovering from COVID-19 − Female athletes recovering from COVID-19 were compared with a group of healthy athletes and a group of healthy controls.	COVID-19 diagnosis-CMR: 17 (IQR 17–19).	-	− Cardiac abnormalities: 0% − No differences between COVID-positive athletes and control athletes
Malek et al. Journal of magnetic resonance imaging, 2021 [20]	Retrospective cohort study	26 consecutive elite athletes (median age 24 years, 21–27; 81% female).	− PE, ECG, and blood tests, including Hs-cTnT and CMR.	COVID-19 diagnosis-CMR: 32 days (IQR 22–62 days).	-	− Myocarditis: 0%. − 19% (5 cases) of cardiac abnormalities at CMR: 4 borderline signs of isolated myocardial edema and one non-ischemic LGE and pleuro-pericardial effusion.
Gervasi et al. Br J Sports Med, 2021 [21]	Prospective cohort study	30 professional soccer players returning to play after the lockdown period [COVID-19-positive group, age 22 (21–27 years]; COVID-19-negative group age 25 (19.5–26.5 years)].	− All athletes: PCR on NP swab and search for SARS-CoV-2 IgG antibodies. − Athletes with evidence of previous COVD-19 infection: PE, ECG, exercise stress testing, TTE, blood testing (including troponin), 24-h ECG monitoring, and a chest CT.	-	-	− No cardiovascular abnormalities; − One player showed increased troponin I without other signs of myocardial damage.
Guevarra et al. Journal of Clinical and Translational Research, 2022 [22]	Retrospective cohort study	99 collegiate athletes (mean age 19.9 ± 1.7 years, 68% male).	− All athletes: a 12 lead ECG after healing. Cardiac troponin T assay, TTE, and CMR performed based on the severity of symptoms.	COVID-19 diagnosis-evaluation: 15 days.	-	− Abnormal post-infection ECG rate: 1%; − No other markers of cardiac involvement observed at echocardiogram and CMR.
Mascia et al. International Journal of Cardiology, 2021 [23]	Retrospective cohort study	58 male soccer players	− COVID-positive athletes: ECG, TTE, Hs-cTnI, CPET, and 24 h ECG Holter monitoring − COVID-19-negative athletes: Hs-cTnI and ECG. − All athletes: CMR if there is a high Hs-cTnI.	COVID-19 diagnosis-CMR: 27–41 days.	-	− COVID-positive group: two subjects with high hs-cTnI − COVID-negative group: two subjects with high hs-cTnI; − All athletes with high hs-cTnI underwent CMR that was unremarkable.
Hendrikson et al. Circulation, 2021 [24]	Retrospective cohort study	137 collegiate (median age 20 years, 18–27; 68% male)	− ECG, echocardiogram, and cTnI − Athletes with any abnormalities underwent CMR.	COVID-19 diagnosis-evaluation: 10 days.	Clinical follow-up: 143 days, IQR 113–239.	− 2.9% of small pericardial effusion at TTE; − 2.9% of high cTnI; − 3.6% of athletes with abnormal testing underwent CMR − CMR was unremarkable for all athletes − At clinical follow-up, no athlete had new symptoms or problems after resuming exercise or during competition
Starekova et al. JAMA, 2021 [25]	Retrospective study	145 competitive student athletes (mean age 20 years, 17–23; 37% female).	− All athletes underwent CMR after healing. Data on ECG, TTEs, blood tests, and clinical aspects were also obtained.	COVID-19 diagnosis-CMR: 15 days (IQR 11–194).	Athletes diagnosed with myocarditis had a clinical and CMR follow-up after 1 month.	− Myocarditis: 1.4% (2 athletes). − 2 abnormal troponin I values without other remarkable cardiovascular findings; − At one month follow-up: one athlete had persistent non-ischemic LGE and another showed a complete resolution of CMR abnormalities.
Szabo’ et al. Br J Sports Med., 2021 [26]	Prospective observational study	147 highly trained athletes (median age 23 years, IQR 20–28 years; 94% male)	− All athletes: Troponin I, ECG, and CMR − Two groups of controls (athletic and non-athletic) underwent CMR. None of the healthy, less active controls underwent contrast administration.	COVID-19 diagnosis-CMR: median 32 days; other examinations recorded at a median of 1 day prior to CMR.	Clinical follow-up at a median of 232 days after the infection.	− Myocarditis: 1.4% (2 athletes). − Other isolated abnormalities at CMR: 4.7% (non-ischemic LGE, elevated native T1 values, pericardial involvement); − 4.5%: high HsTnT (only one patient with abnormal CMR) − No differences in CMR parameters between the different groups; − 83% of athletes underwent follow-up: only two athletes did not return to their sports activity, one due to the progression of depression and one due to Long-COVID syndrome; − 4 athletes underwent CMR follow-up: abnormalities disappeared in three patients.
Clark et al. Circulation, 2021 [27]	Retrospective study	59 collegiate athletes (median age 20 years, 19–21; 63% female)	− All athletes: Troponin I, ECG, TTE (with strain analysis), and CMR. − 60 athletic controls.	COVID-19 diagnosis-CMR: 21.5 days (IQR 13-37).	-	− Myocarditis: 3% (2 athletes); − Pericarditis: 1.7% (1 athlete); − Mild isolated increase in T1 mapping, T2 mapping, or extracellular volume: 39% of COVID-19-positive athletes, 13% of athletic controls, and 8% of healthy controls.
Maestrini et al. Journal of clinical medicine, 2021 [28]	Prospective observational study	47 consecutive enrolled Olympic athletes (mean age 26 ± 4 years, 68% male)	− All athletes: PE, blood tests, ECG, TTE, pulmonary function tests, CPET, 24-h ECG monitoring, and CMR. − All athletes had a previous evaluation (Olympic pre-participation screening program); a subgroup also performed previous CMR.	Negative swab-evaluation: 9 days (6–13).	Athletes with cardiac abnormalities were evaluated after 3 months of sport withdrawal.	− Myocarditis: 2% (1 case). − 6% (3 cases) of new cardiac abnormalities after COVID-19: new pericardial effusion, acute myocarditis, complex PVCs with unremarkable echocardiogram, and CMR; − After three months of sport withdrawal: unremarkable echoes and CMR but still uncommon ventricular arrhythmias − No significant differences between the pre- and post-infection CMR parameters in a subgroup of 18 athletes.
Maestrini et al. Journal of Science and Medicine in Sport, 2022 [29]	Cross sectional study	219 consecutive athletes (median age 23 years, IQR 19–27; 59% male)	− All athletes prior to RTP: PE, ECG, blood test, CPET, 24-h ECG monitoring, spirometry, and CMR on clinical indication.	Negative swab-evaluation: 10 days (IQR 6–17). COVID-19 diagnosis-CMR: 16 days (IQR 13–24).	Athletes with cardiac abnormalities were evaluated after 3 months of sport withdrawal.	− Myocarditis: 0.9% (2 cases). − Pericardial effusion (isolated): 0.45 % (1 case). − Uncommon PVCs: 9.5% at CPET/24 h Holter ECG monitoring. − Athletes temporally withdrawn from sport: 2% (5 cases: 2 myocarditis, 2 heavy burden of uncommon PVCs, 1 exercise-induced PVC with R on T and couplets). − At follow-up: disappearance of abnormalities in only 2 cases (heavy burden uncommon PVCs).
Cavigli et al. International Journal of Cardiology, 2021 [30]	Prospective study	90 professional and non-professional competitive athletes (mean age 24 ± 10 years, 71.1% male)	− All athletes: PE, blood testing, spirometry, 12-lead resting ECG, 24-h ambulatory ECG monitoring, echocardiogram, and CPET. − Additional investigations (CMR and CT) were performed on clinical indication.	Negative swab-evaluation: 15 to 30 days.	Athlete with myocarditis was evaluated after 3 months of sport withdrawal.	− Pericarditis: 3.3% (3 athletes). − Myo-pericarditis: 1.1% (1 athlete). − At three months follow up: resolution of myocardial involvement.
Erickson J et al. Mayo Clinic Proceedings Innovation and quality outcomes, 2021 [31]	Retrospective cohort study	170 collegiate athletes (ages 18–25 years, 91% male)	− All athletes: general examination and ECG. Further examinations if there are abnormal findings. − Athletes divided based on sex, symptom severity, and BMI.	COVID-19 diagnosis-cardiac screening: 22.54 ± 14.20 days.	-	− 3.5% of abnormal ECG findings; − 2 post-COVID effusive viral pericarditis and 1 xiphoiditis.
Moulson et al. Br J Sports Med, 2022 [38]	Prospective observational study	21 consecutive young athletes (mean age 21.9 ± 3.9 years, 43% female)	− All athletes with cardio-pulmonary symptoms lasting more than 28 days from COVID-19 diagnosis underwent a comprehensive evaluation (ECG, Hs-Tn, TTE, CPET, and CMR on clinical indication). A subgroup repeated CPET between 3 and 6 months after the initial evaluation.	COVID-19 diagnosis-cardiac evaluation: 3.0 ± 2.1 months.	6 months	− Active inflammatory heart disease: 0%. − Symptoms reproduced at CPET: 86% of athletes − COVID-19-positive athletes: a more abnormal spirometry (42%) and breathing reserve (42%) than the athletic control group (*n* = 42); − CPET follow-up (13 athletes): 69% reduction in symptoms, improvement in peak vO2 and oxygen pulse, and reduction in resting and peak heart rate.
Multi-center Studies						
Moulson et al. Circulation, 2021 [32]	Prospective observational cohort study	3018 collegiate athletes from the ORCCA registry (mean age 20 ± 1 years, 32% female)	− Analysis of de-identified data from athletes with SARS-CoV-2 infection who completed cardiac evaluation (ECG, cardiac troponin, TTE, and CMR). − Definite, probable, or possible myocardial involvement: the definition is based on CMR criteria and supportive findings (TTE and troponin).	COVID-19 diagnosis-Cardiac tests: − ECG: 12 days (IQR 10–16) − TTE: 15 days (IQR 11–25) − cTn: 12 days (IQR 10–17) − CMR: 33 days (IQR 18–63).	Clinical follow-up among the entire cohort: 113 days (IQR 90–146). Clinical follow-up among athletes with SARS-CoV-2 cardiac involvement (definite, probable, or possible): median of 130 days (IQR 97–160)	− 13% of athletes: cardio-pulmonary symptoms during the infection or upon return to exercise − 198 athletes underwent CMR irrespective of symptoms or results of other testing − 119 underwent CMR on clinical indications; − 0.7% (21 athletes) with definite, probable, or possible SARS-CoV-2 cardiac involvement − Diagnostic yield of CMR for SARS-CoV-2 cardiac involvement: 4.2 times higher for a clinically indicated CMR (15 of 119 [12.6%]) versus a primary screening CMR (6 of 198 [3.0%]) − On multivariable analysis: cardio-pulmonary symptoms and any abnormal triad test results were predictive of cardiac involvement − At follow-up: 1 (0.03%) adverse cardiac event, likely unrelated to SARS-CoV-2 infection and no adverse cardiac events in athletes with SARS-CoV-2 cardiac involvement
Daniels et al. Jama Cardiology, 2021 [33]	Observational Study	1597 athletes (60% male) from 13 universities, participating in the Big 10 COVID-19 Cardiac Registry	− Analysis of de-identified data from athletes with SARS-CoV-2 infection who completed a cardiac evaluation (ECG, cardiac troponin, TTE, and CMR). − Data on age and race were not collected.	COVID-19 diagnosis-cardiac testing: 22 days (range 10–78 days).	CMR follow-up in athletes with myocarditis diagnosis: 9.4 ± 3.1 weeks.	− Myocarditis prevalence: 2.3% (37 athletes) − Clinical myocarditis reporting cardiac symptoms (chest pain, dyspnoea, and palpitations): 9 athletes − Subclinical myocarditis (no cardiac symptoms): 28 athletes, 8 with abnormal cardiac testing and 20 diagnosed only by CMR imaging; − CMR imaging: increased prevalence of 7.4-fold over the symptoms driven strategy and 2.8-fold over the ECG/echo and troponin strategy; − CMR follow up (27 athletes, 73%): 40.7% complete resolution and 59.3% T2 mapping abnormalities resolution with persistence of LGE.
Martinez et al. JAMA, 2021 [34]	Cross sectional study	789 professional athletes (mean age 25 ± 3 years, 98.5% male)	− Analysis of de-identified data from athletes with SARS-CoV-2 infection who completed cardiac evaluation (troponin testing, ECG, TTE, CMR and/or stress echocardiography on clinical indication).	COVID-19 diagnosis-cardiac testing: 19 ± 17 days (range of 3–156).	Clinical follow-up: from enrollment (May-October 2020) to December 2020.	− Abnormal screening results: 3.8% (30 athletes, 0.8% troponin, 1.3% ECG, 2.5% TTE) − At CMR: 0.6% (5 athletes) findings suggestive for inflammatory heart disease at CMR (3 myocarditis; 2 pericarditis) − No adverse cardiac events occurred in athletes who underwent cardiac screening and resumed professional sport participation
Chevalier et al. Sports Medicine—Open, 2022 [35]	Prospective cohort study	950 athletes: 779 professional league rugby players (mean age 25.8 ± 4.6, 100% male) and 171 student athletes (mean age 20.1 ± 3.1, 49.7% male)	− All athletes: ECG and biomarkers profiling at baseline. − COVID-19-positive athletes (if history of COVID-19 at inclusion for both athletes’ categories or developing the infection during the study for professional athletes): ECG, TTE, and stress testing at baseline and before re-starting physical activity. CMR was offered to all enrolled professional and student athletes.	COVID-19 diagnosis-CMR: 51 ± 37 days. Healing-lab tests: 7 (7–13) days.	Clinical follow-up: 289 ± 56 days.	− No baseline ECG abnormalities were found − Abnormal ECG: 2.6% (6/234) of COVID-positive athletes − Abnormal TTE: 0.4% (1/233) of COVID-positive athletes (new regional wall motion abnormality) − Stress test abnormalities: 4.3% (10/231) of COVID-positive athletes (isolated ventricular ectopy) − CMR performed in 130 athletes (102 COVID-positive and 28 COVID-negative); − LGE: 1.6% (2 athletes, one from the COVID-positive and one from the COVID-negative) − Myocarditis: 0% − At follow-up: one adverse event (ventricular tachycardia during a match in an athlete in the COVID-positive group with a history of infection 64 days prior to the event and a normal post-infection comprehensive cardiovascular evaluation).
Casasco et al. Journal of Cardiovascular Development and Disease, 2022 [36]	Prospective observational study	4143 athletes (mean age 22.5 ± 13.3 years, ranging from 8 to 80 years; 67.8% male)	− Analysis of data from athletes undergoing the Italian RTP protocol after COVID-19: − Class 1 (asymptomatic/mild symptomatic athletes): resting ECG and ECG during maximal incremental exercise testing with continuous O_2_ saturation monitoring and TTE. − Class 2 (moderate disease or hospitalization): blood tests, 24-h ECG monitoring, and respiratory function assessment in addition. − Class 3 (severe or critical disease): CPET in addition. − CMR performed upon clinical indication. − Professional athletes considered as class 3.	Healing-cardiac testing: 30 days.	-	− Abnormal TTE findings: 1.9% of athletes − Arrhythmic events: 5.4% during exercise testing and 0.4% during 24 h ECG monitoring; − Ventricular arrhythmias at exercise testing or 24-h ECG monitoring: 2.4% of athletes − CMR performed in 34 athletes − Myocarditis: 0.12% of the total population − Pericarditis: 0.2% of athletes
Hedon et al. Arch Cardiovasc Dis, 2022 [37]	Retrospective cohort study	554 high-level athletes (professional or national level; median age 22 years, 72% male)	− Analysis of de-identified data from athletes with SARS-CoV-2 infection who completed cardiac evaluation: (ECG, TTE, exercise stress, 24-h ECG monitoring, and troponin T).	-	Clinical follow-up: 376 ± 125 days.	− Anomalies with a potential link with SARS-CoV-2 infection: 0.7% ECGs (4/551), 0.6% TTEs (3/497), and 1.0% exercise tests (1/293); − Cardiac troponin elevation: 1.1% (one athlete with unremarkable further examinations) − CMR abnormalities: 2.9% (1/34). − Possible SARS-CoV-2 myocarditis: one athlete (unknown ECG repolarization anomalies and inferolateral LGE at CMR) − At follow-up: 0% of major cardiac events; − No differences in cardiovascular examination between groups with different symptom intensities.
Petek et al. Br J Sports Med, 2022 [39]	Prospective observational cohort study	3644 collegiate athletes from the ORCCA registry (mean age 20 years, 34% female)	− Analysis of de-identified data from athletes with SARS-CoV-2 infection and persistent/exertional symptoms who underwent a comprehensive evaluation (CMR, cardiac stress testing, CPET, chest X-ray, coronary CT angiography, CT-PE, ambulatory Holter/event monitoring, or pulmonary function testing).	COVID-19 diagnosis-RTP: 17 days (IQR 13–21). COVID 19 diagnosis-CMR: 44 days (IQR 29–70).	Clinical follow-up: at least 12 weeks.	− Persistent symptoms > 3 weeks: 1.2% athletes − Persistent symptoms > 12 weeks: 0.06% − Exertional cardio-pulmonary symptoms: 4.0% athletes − Diagnosis of SARS-CoV-2-associated sequelae: 0% of athletes with isolated persistent symptoms, 8.8% athletes with exertional symptoms (5 cardiac involvement, 2 pneumonia, 2 inappropriate sinus tachycardia, 2 postural orthostatic tachycardia syndrome, and 1 pleural effusion). − Probable or definite cardiac involvement: 20.8% of athletes with exercise chest pain who underwent CMR (5/24), 0% of athletes with exertional symptoms other than chest pain
Petek et al. Circulation, 2022 [40]	Prospective observational cohort study	3675 collegiate athletes from the ORCCA registry (mean age 20 ± 1 years, 33% female)	− Analysis of data from athletes recovering from COVID-19 who underwent cardiovascular evaluation (ECG, troponin, TTE, or CMR) and had follow-up from the participating institution.	-	Median follow-up: 1.12 years (IQR 1.06–1.22).	− Definite or probable SARS-CoV-2 myocardial or myo-pericardial involvement: 0.6% − They were restricted from the sport and were successfully returned to the sport after a median of 86 days (IQR 33–90) − Follow-up cardiac imaging: 71% athletes − Follow-up CMR: 70% complete resolution, 10% partial resolution, and 20% persistent CMR abnormalities − Clinical follow-up: 0.05% (2 cases) adverse cardiovascular events, both in athletes without SARS-CoV-2 cardiac involvement

## Data Availability

Data sharing not applicable.

## Abbreviations

BMI	body mass index
BNP	brain natriuretic peptide
CMR	cardiac magnetic resonance
COVID-19	coronavirus disease 2019
CPET	cardio-pulmonary exercise test
CRP	C reactive protein
CT	computed tomography
cTn	cardiac troponin
CT-PE	computed tomography
ECG	electrocardiogram
GLS	global longitudinal strain
Hs-Tn	high sensitive cardiac troponin
IgG	immunoglobulin G
IQR	interquartile range
LGE	late gadolinium enhancement
NP	naso-pharyngeal
ORCCA	outcome registry for cardiac conditions in athletes
PCR	polymerase chain reaction
PE	physical examination
PVCs	premature ventricular complexes
RTP	return to play
SARS-CoV-2	severe acute respiratory syndrome coronavirus 2
TTE	transthoracic echocardiography
